# The prospective roles of exosomes in pituitary tumors

**DOI:** 10.3389/fendo.2024.1482756

**Published:** 2024-11-22

**Authors:** Paulina Lisiewicz, Małgorzata Szelachowska, Adam Jacek Krętowski, Katarzyna Siewko

**Affiliations:** Department of Endocrinology, Diabetology and Internal Medicine, Medical University of Bialystok, Bialystok, Poland

**Keywords:** PitNET, pituitary adenoma, pituitary tumor, exosomes, extracellular vesicles, miRNA, proteome

## Abstract

Pituitary neuroendocrine tumors are common, typically benign intracranial neoplasms arising from well-differentiated anterior pituitary cells with prevalence of clinically relevant pituitary tumor of 89 in 100 000 people. Despite the growing number of published studies, there is still a need for diagnostic and predictive biomarkers of pituitary adenomas. Prompt determination of tendency of the tumor for invasive growth and aggressive behavior would allow for earlier and more effective treatment. Extracellular vesicles (EVs), including exosomes, are particles released by cells containing cell-specific cargo including a variety of bioactive molecules, such as DNA, messenger RNA, microRNA, long non-coding RNA, circular RNA, proteins, and lipids surrounded by lipid membranes, which act as mediators of cell to cell communication. The ability of exosomes to reflect the functional state of the tumor, transport informative molecules, and accessibility in body fluids make them promising candidates in the search for biomarkers and new therapeutic methods. This study aims to investigate the involvement of exosomes in the pathology of pituitary adenoma and their potential clinical applications.

## Highlights

- Exosomes are cell crosstalk mediators that contain bioactive molecules and are easily accessible in body fluids; thus, they are promising as novel biomarkers.
- Exosomal micro RNA, circular RNA, long non-coding RNA and messenger RNA expression were altered in pituitary adenomas and in some studies were correlated with tumor invasiveness, diagnosis, and prognosis.
- Functional studies suggest that exosomal RNA may alter the tumorigenesis of pituitary adenomas or other tumor cells and can be regarded as a therapeutic agent.
- Alterations in exosomal protein expression between invasive and non-invasive pituitary adenomas were identified and proposed as potential liquid biopsy markers of the tumor invasiveness.
- Further evidence on the role of exosomes in pituitary adenomas is required.

## 1 Introduction

Pituitary neuroendocrine tumors are typically benign intracranial neoplasms arising from well-differentiated anterior pituitary cells, with a prevalence in the general population ranging from approximately 10% in autopsy studies to 22% in radiological studies, and with a prevalence of clinically relevant pituitary tumor of 89 in 100 000 people (1). The clinical picture of the disease may vary from asymptomatic in small and non-functioning pituitary neuroendocrine tumors to symptoms and signs resulting from hypersecretion of a hormonally active tumor, or, in the case of larger tumors (typically >1 cm), from its mass effect on the surrounding structures. This mass effect can lead to hypopituitarism, headaches or visual field defects, which can cause severe disabilities (2, 3).

Pituitary neuroendocrine tumors are classified into non-functioning (57%) or hormonally active (43%) secreting pituitary hormones, such as growth hormone (GH) -18%, prolactin (PRL)-12%, adrenocorticotrophic hormone (ACTH)-5%, gonadotropins (luteinizing hormone (LH), follicle-stimulating hormone (FSH) and alpha-subunit of glycoproteins -5%, thyroid-stimulating hormone (TSH)-1%, or mixed tumors most commonly secreting growth hormone and prolactin -1% (4). The epidemiology of the tumor types varies between studies, with non-functioning pituitary neuroendocrine tumors and prolactinomas being the most common with a prevalence of 14-43% and 31-66% respectively (5).

In 2017, the World Health Organization (WHO) introduced a new classification of pituitary tumors that distinguishes them not only by hormonal activity but also by transcription factors, such as: pituitary-specific positive transcription factor 1 (PIT1), T-box transcription factor (TPIT) and steroidogenic factor 1 (SF1). The PIT1 lineage is associated with somatotroph, lactotroph, mammosomatotroph, mature plurihormonal, immature, and acidophil stem cell tumors, the TPIT lineage with corticotroph tumors, the SF1 lineage with gonadotroph tumors, and the category of pituitary neuroendocrine tumors with no distinct cell lineage, including null cell tumors and plurihormonal tumors (**Table 1**) (6, 7). However, there is disagreement as to the clinical usefulness of this classification because 1) it cannot be applied to pituitary tumors that were not treated surgically; 2) it does not include clinical, radiological, biochemical, or genetic information; and 3) it does not propose any grading or staging system (8, 9).

**Table 1:** The 5^th^ WHO classification of pituitary neuroendocrine tumors/pituitary adenomas.

Lineage	Pituitary neuroendocrine tumor type and subtype	
PIT1-lineage	Somatotroph tumors	Densely granulated somatotroph tumor
		Sparsely granulated somatotroph tumor
	Lactotroph tumors	Densely granulated lactotroph tumor
		Sparsely granulated lactotroph tumor
	Mammosomatotroph tumor	
	Thyrotroph tumor	
	Mature plurihormonal PIT1-lineage tumor	
	Immature PIT1-lineage tumor	
	Acidophil stem cell tumor	
	Mixed somatotroph and lactotroph tumor	
TPIT-lineage	Corticotroph tumors	Densely granulated corticotroph tumor
		Sparsely granulated corticotroph tumor
		Crooke cell corticotroph tumor
SF1-lineage	Gonadotroph tumor	
No distinct cell lineage	Plurihormonal tumor	
	Null cell tumor	

Transsphenoidal pituitary surgery is the first-line treatment for clinically significant pituitary adenomas, excluding prolactinomas. Other treatment options for pituitary adenomas include radiation therapy and pituitary-directed medical therapy using somatostatin receptor ligands or dopamine agonists in tumors positive for somatostatin receptor 2, somatostatin receptor 5, or dopamine D2 receptor respectively. Usually, non-functioning and asymptomatic pituitary adenomas are managed conservatively. Nevertheless, the presence of symptoms and signs related to the tumor placing pressure on surrounding structures, which is typical of large tumors, is an indication for surgery (3, 10).

The outcomes of pituitary adenoma surgery presented in a meta-analysis and survey study in 2022 indicated remission rates of 50% for acromegaly, 68% for Cushing’s disease, and 53% for prolactinoma. Gross total resection was achieved in 49% of patients with no difference between non-functioning or hormone-producing adenomas (11).

According to the literature, approximately 45-55% of pituitary adenomas invade local structures such as the cavernous sinus, sphenoid, or dura mater. Along with tumor size, local invasion is one of the main factors contributing to an incomplete outcome of pituitary surgery, resulting in a higher risk of persistent disease. One observational study examined 354 patients with pituitary adenoma who underwent transsphenoidal surgery. Residual disease was demonstrable in 14-17% of patients with non-invasive pituitary adenoma and 30-50% of patients with invasive pituitary adenoma. The survival rate was lower in patients with invasive pituitary adenoma (91%) than those with non-invasive pituitary adenoma (100%) at six years post-surgery (12). Another case-control study comprising 410 patients with pituitary adenoma reported that the grade of tumor invasion was associated with disease progression or recurrence at eight years post-surgery (13).

Although mostly slow-progressing and benign, 0,5% of pituitary tumors may have more aggressive behavior, such as local invasion, rapid growth, and poor response to the standard treatment, which prompted a change of nomenclature in the aforementioned WHO 2017 classification from “pituitary adenoma” to “pituitary neuroendocrine tumor”, because the term adenoma, by definition, describes benign tumors (6, 14). Nevertheless, this claim is disputed, suggesting that the biology of pituitary tumors differs from that of most neuroendocrine tumors and that this change in nomenclature would increase anxiety and cause unnecessary confusion (15). Both forms of the nomenclature can be found in the literature.

Classical markers of aggressive behavior in pituitary tumors, such as a high ki-67 index and extensive p53 staining, are not sufficiently accurate because they do not discriminate between approximately 20% of aggressive tumors, even though they are linked to a higher risk of recurrence (16).

Despite the growing number of studies in this field, the currently available diagnostic and prognostic parameters based on tumor imaging, histopathology and biochemical and molecular markers do not fulfil clinical requirements, particularly in terms of assessing tumor invasiveness and aggressiveness. There is still a demand for tools that will assist in providing a more accurate diagnosis and non-invasive diagnosis and prognosis that would potentially aggressive and invasive tumors to be identified, leading to earlier radical treatment, thus minimizing the risk of persistent or recurrent disease.

Increasing attention has been drawn to the role of exosomes, small extracellular vesicles (EVs), as potential tumor biomarkers that may reflect tumor characteristics and that can be accessed in a non-invasive manner in virtually all body fluids. Owing to their unique properties, exosomes mirror the origin cell state, transport bioactive molecules, participate in cellular crosstalk, and influence recipient cell behavior, possibly facilitating tumor proliferation, invasiveness, and recurrence. Clinical trials are conducted to investigate the clinical applications of exosomes for use as biomarkers, exosome therapy, drug-delivery systems or cancer vaccines in various pathologies (17–19).

This study aimed to investigate the involvement of exosomes in the pathology of pituitary adenoma and their potential clinical applications.

## 2 Extracellular vesicles and exosomes – structure, composition, and role

EVs are surrounded by lipid membrane particles released by cells. Thay were initially described in 1967 as procoagulant “platelet dust” and since then they have become known as mediators of cell- to- cell communication (20). They can be found in peripheral blood plasma and serum at a concentration >10^9^/ml, whereas up to 25-75% of blood EVs originate from platelets. EVs have also been found in other body fluids such as urine, tears, saliva, cerebrospinal fluid, bile, nasal and bronchial lavage, amniotic fluid, breast milk, and seminal fluid (21, 22). In 2006-2007 microRNA (miRNA) and messenger RNA (mRNA) were found in EVs, drawing attention to their role in the direct and safe transport of informative molecules (23–25).

Based on their size and biogenesis, EVs have been divided into three main subtypes: microparticles or microvesicles, apoptotic bodies and exosomes (21). The largest of them, apoptotic bodies are 50 to 5,000 nm in size, contain mostly DNA, and originate from cell fragmentation during apoptosis (26). Microvesicles measuring 50 to 1,000 nm are released from cells via cell membrane budding and scission; therefore, they mostly contain phosphatidylserine and membrane proteins (27). Recent studies have described new classes of EVs that are generated by the scission of cellular protrusive structures, such as filopodia, microvilli, and cilia, and may share dimensions and markers such as tetraspanins with other EV types, signaling the requirement to adapt the classification criteria (28–30).

Exosomes, described as 30-100 nm in size, extracellular, membranous, cup or dish- shaped structures formed in intracellular multivesicular bodies and released through exocytosis, were found to originate from most cell types. However, their size, composition, and genesis vary not only between cell types but also within individual cells, which may release a variety of exosomes depending on their functional state due to selective sorting of the cargo under the control of an endocytosis sorting complex. The process of exosome formation includes invagination of the cell membrane and creation of sorting endosomes that form multivesicular bodies that release exosomes by fusion with the cell membrane. The main pathway of multivesicular body formation is the endosomal sorting complex required for transport (ESCRT)-dependent pathway but ESCRT-independent mechanisms have also been described. However, the exact mechanisms underlying exosome formation and secretion remain unclear (31, 32).

Protected by a lipid membrane covered with markers, exosomal cargo may include a variety of molecules, such as single-stranded DNA, double-stranded DNA, amplified oncogene sequences, mitochondrial DNA, mRNA, miRNA, lncRNA, circRNA, proteins, and lipids (33–37). Tetraspanins (CD9, CD63, CD81, CD82, CD151, and Tspan8), class I and II histocompatibility complexes, adhesion molecules, proteases, and the heat-shock proteins HSP 60 and HSP70 are consistent components of exosome cargo (38).

**Figure 1:**
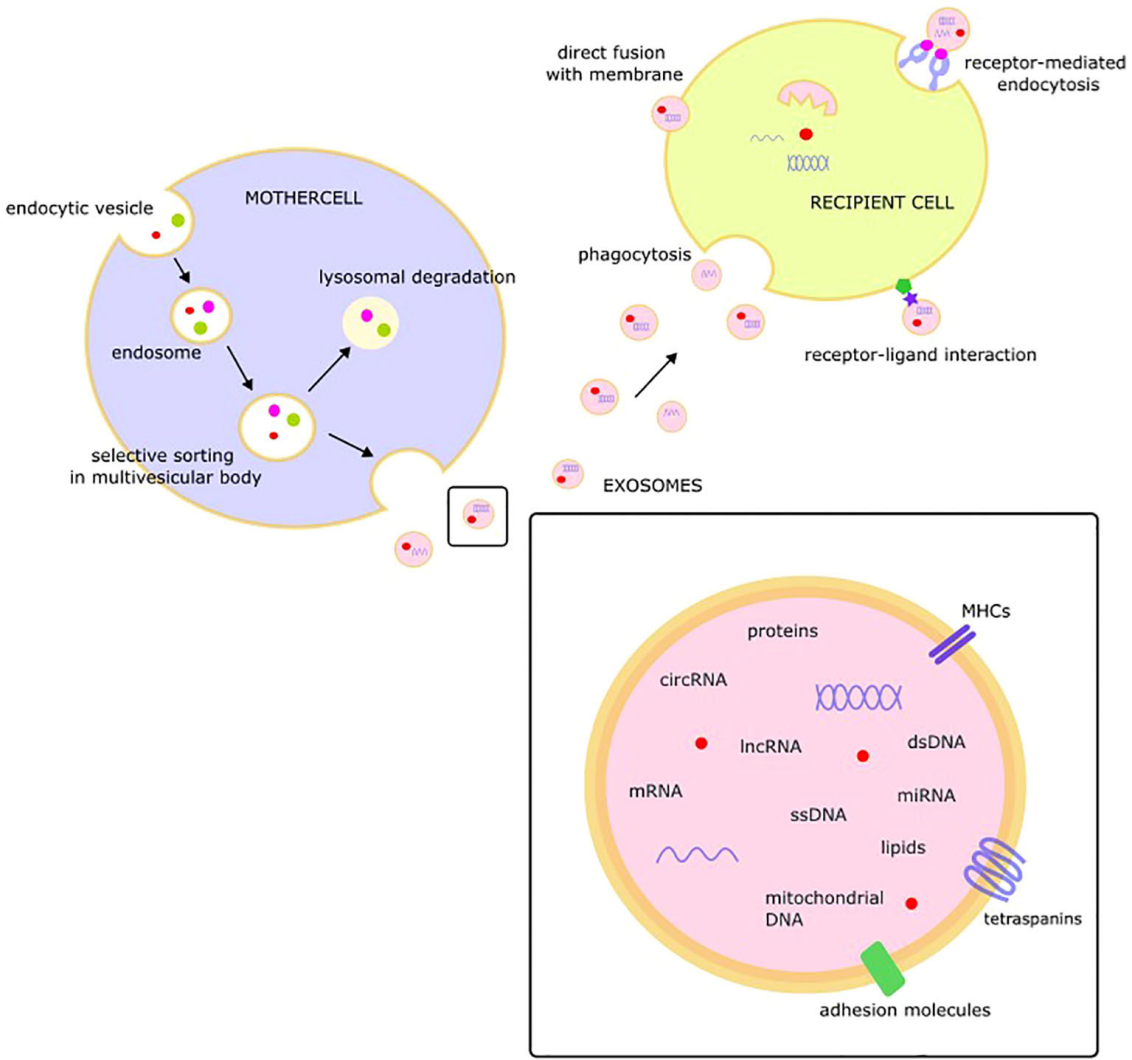
The structure, composition, and role of exosome.

The released exosomes may interact with target cells and change their behavior via fusion with the membrane of the target cell, receptor-mediated endocytosis, receptor-ligand interactions, or phagocytosis (39). After fusion with the recipient cell membrane, exosomal cargo, including bioactive molecules such as miRNA, proteins, or lipids, is released into the cytoplasm of the target cell. This “language of exosomes” may be viewed as a novel type of intercellular communication with EVs as endocrine/paracrine messengers (**Figure 1**) (40).

## 3 EVs – methods of isolation and analysis

New methods for the extraction and analysis of EVs have been developed as a relatively new area of focus. The International Society for Extracellular Vesicles formulated general guidelines for conducting studies on EVs (41).

EVs are difficult to isolate owing to their heterogeneity in size, content, and source. Ultracentrifugation is one of the most popular techniques for this purpose. However, the isolation and purification of exosomes from biological fluids requires a combination of different techniques, such as density gradient centrifugation, filtration, precipitation, size-exclusion chromatography, or liquid chromatography. The polyethylene glycol-dextran aqueous two-phase system is another interesting method for EV isolation from body fluids containing high quantities of proteins as it has high recovery efficiency and purity of isolated EVs (42).

Plasma is the preferred material for isolating EVs from the blood. In serum samples, over 50% of the EVs were released from clotting platelets (43). Heparin is the only anticoagulant that should not be used as it may bind to the EVs (44). Cells should be removed from any source material as early as possible because cell disruption, post-collection cellular activation, and cell death may influence EV composition and function. The collected material with cells and platelets removed can be stored using freezing, freeze-drying, or drying techniques for protection. Phosphorylation proteins can be isolated from frozen samples stored for five years. Nevertheless, more data are required to evaluate long-term storage stability (45).

Characterization techniques include western blotting, nano particle tracking analysis, flow cytometry, and electron microscopy (46). Markers of EVs can be divided into three categories: membrane markers such as CD63, CD81, CD82, CD47, MHC class I, and integrins -ITGA*/ITGB*; cytosolic markers, such as ESCRT-I/II/III, ALIX, VPS4A/B, ARRDC1, Flotillins-1 and 2, heat shock proteins HSC70 and HSP84; and negative markers such as apolipoproteins A1/2 and B or albumin for plasma/serum- derived EVs (47).

Hoshino et al. conducted a proteomic analysis of EVs and EV particles (EVP) derived from 426 human tissue, plasma and body fluid samples and validated CD9, HSPA8, ALIX, and HSP90AB1 as pan-EVP markers while suggesting ACTB, MSN, and RAP1B as novel pan-EVP markers. This study also identified candidate markers for EVP isolation, cancer detection, and determining cancer type (48).

There are a few EV databases such as EVpedia, Vesiclepedia and Exocarta that provide a source of information on EV research and their components in different types of pathologies; however, the data provided may not be regularly updated (49–54).

## 4 Clinical potential of exosomes

EVs are produced by virtually all cells and are found in all body fluids. The composition of EVs reflects the state of their cell of origin. Therefore, profiling the contents of their cargo, such as miRNA, proteins, or lipids, may result in the identification of new markers that may assist in the diagnosis or monitoring of disease using minimally invasive liquid biopsy. Cancer cells produce a large number of EVs that participate in tumor development. Neoplastic cells produce 10-fold more EVs than normal cells. Molecules of exosomal origin facilitate angiogenesis, epithelial-mesenchymal transition, formation of premetastatic niche, cancer cell immune escape and drug resistance (48, 55–60).

In addition, owing to their characteristic property of transporting cargo to target cells, exosomes are viewed as a promising method of precisely delivering therapeutic agents precisely to target cells, safely protected from degradation by the lipid bilayer, and resistant to uptake by the immunological system. A limitation of this method is the rapid clearance of exosomes, which necessitates further exosome engineering (61–63).

More than 400 studies on exosomal biomarkers and treatment methods are being conducted, and they can be found at clinicaltrials.gov (64).

## 5 Genetic analysis of exosomes in search of pituitary adenoma biomarkers

### 5.1 miRNA

miRNA has been widely studied in pituitary adenomas. miRNA is a non-coding, short RNA molecule that regulates gene expression at the post-transcriptional level by binding to the 3′-untranslated region of target mRNA, inhibiting gene translation or causing mRNA degradation (65) miRNAs are found not only within cells but also in body fluids and are referred to as circulating miRNAs. To protect against RNase degradation, circulating miRNAs are encapsulated in extracellular vesicles or circulate in a non-vesicle- associated form as ribonucleoprotein complexes (66). Arroyo et al. suggested that up to 90% of circulating miRNAs are the non-vesicle- associated (67). However, some studies have suggested that miRNA expression differs between blood-derived exosomes and plasma samples (68–70).

Tumorigenesis generally results from overexpression of miRNAs targeting tumor suppression genes (oncoMIRs) and suppressed expression of miRNAs that target oncogenes (tumor suppressor miRNAs). Dysregulation of miRNA is considered a tumor hallmark and is tissue specific (71).

Due to their stability in biological fluids and high specificity, circulating miRNAs have been widely studied in the search for novel pituitary adenoma biomarkers. However, most of these studies have not distinguished between miRNA of exosomal origin.

Nemeth et al. examined plasma, plasma exosomal (preoperative, early postoperative, and late postoperative) and tissue samples of 45 patients with pituitary adenomas divided according to hormonal activity and transcription factors: 29 FSH/LH+, seven GH -secreting, three plurihormonal, three hormone-immunonegative steroidogenic factor 1+ (SF1+), and three hormone-immunonegative T-box transcription factor TBX19+ (T-Pit) adenomas finding that all the measurable miRNAs in hormone-immunonegative and somatotroph adenoma plasma samples (miR ‒26b-5p, miR ‒126-5p, miR ‒148b-3p, and miR ‒150-5p) were also detectable in exosomes. Nevertheless, none distinguished between preoperative and postoperative samples (72).

In another study, Lyu et al. investigated the exosomal miRNA profiles of non-functioning pituitary adenomas. Global downregulation of exosomal miRNAs in non-functioning pituitary adenomas compared to healthy controls was revealed, consistent with the previously described global downregulation of miRNAs in pituitary adenoma tissue samples and plasma (72). Exosomal hsa-miR-486-5p, hsa-miR-151a-5p, hsa-miR-652-3p_R+1, and hsa-miR-1180-3p were upregulated in non-functioning pituitary adenoma samples and were proposed as candidate biomarkers for non-functioning pituitary adenoma, with hsa-miR-486-5p being the most efficient biomarker for predicting disease progression or relapse of disease (area under the receiver operating characteristic curve (AUC) = 0.9432). Thus, the authors suggested that high preoperative expression of exosomal hsa-miR-486-5p could be an indication for more radical treatment in clinical settings. Bioinformatics methods indicated that exosomal hsa-miR-486-5p may be involved in the regulation of mitogen-activated protein kinase (MAPK) signaling pathways. However, further investigation is required. Interestingly, no differences in exosomal miRNA expression were found between invasive and non-invasive non-functioning pituitary adenomas (73).

Zhao et al. performed miRNA profiling of exosomes derived from the serum of six patients with somatotroph adenomas and found 169 differentially expressed miRNAs, among which hsa-miR-320a and hsa-miR-423-5p were downregulated. Analysis of mRNA and proteomic targets revealed the overexpression of SYT1 and PTTG1 in somatotroph adenoma tissues, which correlated with tumor size, hormone concentration, and tumor recurrence. *In vitro* experiments showed that miR-423-5p inhibits cell proliferation, induces cell apoptosis, and reduces growth hormone release and migration in GH3 cells (74).

### 5.2 circRNA

circRNA is a novel, tissue-specific, non-coding RNA characterized by a closed-loop structure that provides greater stability and resistance to RNA exonucleases. Emerging circRNA studies have revealed various biological functions showing that they may act as miRNA sponges by binding to miRNAs and thereby regulating their expression. Similarly, circRNAs may bind to proteins and facilitate their transport between the nucleus and cytoplasm, or affect translation by binding to mRNA. Finally, circRNAs may be translated to produce proteins (75).

Yue et al. explored exosomal circCCDC66 expression levels in the serum of 90 patients with pituitary adenoma (28 prolactinoma, 10 adrenocorticotrophic hormone (ACTH)-secreting, 22 GH–secreting and 30 non-functioning pituitary adenomas) pre- and post- surgery, and 50 healthy subjects finding that circCCDC66 expression was increased in serum exosomes and pituitary adenoma tissue of patients with pituitary adenoma compared to healthy controls. Serum exosomal circCCDC66 expression discriminated patients with pituitary adenoma from healthy controls with 80.00% sensitivity and 84.00% specificity. Exosomal circCCDC66 expression was downregulated following pituitary adenoma surgery and increased at the time of recurrence of the disease showing s positive correlation between circCCDC66 expression and tumor size, invasiveness and recurrence (76).

### 5.3 mRNA

Yu et al. compared mRNA expression of candidate genes linked to tumor progression or invasion, including cyclin dependent kinase 6 (*CDK6*), ras homolog family member U (RHOU), and spire type actin nucleation factor 2 (*SPIRE2*) in serum exosomes derived from patients with invasive and non-invasive non-functioning pituitary adenoma. *CDK6* and *RHOU* expression were upregulated in patients with invasive pituitary adenoma with AUCs of 0.77 (95%) and 0. 76 (95%), respectively, suggesting that they have role as markers of tumor invasiveness (77).

Bao et al. analyzed candidate protein expression in 39 patients with non-invasive and 22 patients with invasive non-functioning pituitary adenoma and found that higher *in situ INSM1*, a zinc finger transcription factor, expression is related to the invasive potential of the tumor, and serum exosomal *INSM1* mRNA expression was higher in patients with invasive pituitary adenoma, with an AUC of 0. 72, suggesting that it is a promising diagnostic factor for tumor invasion (78).

Although still in its infancy, the search for exosomal RNA biomarkers of pituitary adenomas and their invasiveness has provided promising candidates. Nevertheless, further studies with larger patient groups are required to validate these findings.

## 6 Functional studies involving exosomal RNA

Exosomes contain microRNA, mRNA or lncRNA that may affect tumorigenesis. Their ability to transport molecules to target cells can be regarded as a part of intercellular communication, indicating their significant role in the pathogenesis of pituitary adenoma and their pivotal role in transporting therapeutic agents to tumor cells.

### 6.1 Exosomal RNA affects pituitary tumorigenesis

In a functional study, Zhao et al. demonstrated decreased expression of miR-149-5p and miR-99a-3p in the rat pituitary tumor cell lines GH3 and MMQ, hypothesizing their role as tumor suppressors; they investigated the effect of exosome- derived miR-149-5p and miR-99a-3p on rat pituitary adenoma cells and found that they inhibit tumor cell proliferation, migration, and invasion. Neuro-oncological ventral antigen 1 (*NOVA1*), denticleless E3 ubiquitin protein ligase homolog (*DTL*), and *RAB27B* were identified as target genes for miR-99a-3p. Nevertheless, further studies are required to establish the expression and function of the target genes, and the exact mechanism through which miR-99a-3p affects progression of pituitary adenoma is yet to be elucidated (79).

In recent years, the emerging role of the tumor microenvironment in pituitary tumorigenesis has drawn considerable attention.

Jiang et al. investigated the role of exosomal circRNAs derived from tumor-associated fibroblasts on the growth of pituitary adenoma. First, the promoting effect of tumor- associated-fibroblasts on pituitary tumor cell growth was demonstrated *in vitro* and *in vivo*. Further analysis demonstrated higher expression levels of circDennd1b in exosomes derived from aggressive pituitary adenoma tissues and exosomes derived from tumor-associated fibroblasts. Furthermore, the circDennd1b level was positively correlated with tumor size and aggressiveness in human pituitary adenoma tissues. Next, circDennd1b was found to promote the progression of pituitary tumor by sponging miR-145-5p, which acts as a tumor suppressor and downregulates the expression of one cut homobox 2, a transcription factor that participates in tumor development by regulating fibroblast growth-factor receptor 3 expression in signaling pathways such as MAPK, mammalian target of rapamycin, and phosphoinositide-3 kinase (80).

Interestingly, pituitary-derived exosomes may also affect other malignant tumor cells.

Zhou et al. analyzed exosomal miRNA and mRNA derived from rat somatotroph tumor cell lines (GH1 and GH3) and tested the actions of GH1-derived exosomes (GH1-exo) (81). Exosomal miRNA and mRNA expression were more specific to pituitary cells than to non-pituitary cells. The GH1-exo action on rat hepatic cells showed decreased mRNA expression of *Eif2ak2* and *Atf4*. This suggests that GH1-exo is involved in amino acid synthesis and metabolic processes. The effect of GH1-exo on malignant HCT116 cells was also examined and GH1-exo and GH1 cells both attenuated HCT116 migration and invasion. Further, mRNA and KEGG pathway analyses indicated that GH1-exo alters several cancer-related pathways such as MAPK or p53 signaling. This study suggests that pituitary-derived exosomes carry the benign properties of mother cells and may attenuate malignant tumor cell migration, invasion and metastasis. GH1 and GH3 cells were treated with hydrocortisone and it was found that hydrocortisone induced exosome release, which is in agreement with other studies, suggesting that stress may enhance exosome release (81–83).

### 6.2 Exosomal RNA participates in the pathogenesis of acromegaly

Xiong et al. conducted a series of experiments using GH adenoma cells, a GH3 cell line, and animal studies to test the hypothesis that exosomes play a role in abnormal bone formation in somatotroph adenomas, independently of the GH/insulin-like growth factor (IGF-1) axis. First, it was demonstrated that exosomes may be internalized by osteoblasts. miRNA analysis of the pituitary tissue showed overexpression of miR-21 in somatotroph adenoma tissue compared to non-functioning adenoma tissue. Treatment with GH3 derived exosomes resulted in increased miR-21 expression in osteoblasts, suggesting the selective sorting and targeted delivery of miRNAs into exosomes. In animal studies, the exposure of rats to GH3 derived exosomes resulted in reduced trabecular separation, which could contribute to increased bone mass. At the molecular level, it was found that GH3 derived exosomes can promote osteoblast proliferation through the miR-21/PDCD4/AP-1 and Smad7/Runx2 pathways and regulate osteoblast differentiation through an increase in collagen I, osteocalcin, and Runx2-expression levels in osteoblasts. These data show that exosomal hsa-miR21-5p participates in the pathogenesis of acromegaly by disturbing the balance between osteoblasts and osteoclasts, leading to abnormal bone growth, independent of the GH/IGF-1 axis and it may become a novel therapeutic target (84).

### 6.3 Exosomal RNA may be regarded as a therapeutic agent

lncRNAs are a class of noncoding RNA molecules longer than 200 nucleotides that regulate gene expression in multiple ways, including acting as competitive endogenous RNA for miRNA (sequestering miRNA), interacting with proteins involved in RNA transcription, or affecting epigenetic regulation (85). Based on the recent finding that lncRNA H19 is downregulated in pituitary adenomas and that its overexpression may inhibit the growth of pituitary tumor cells, Zhang et al. investigated whole blood- derived exosomes in 206 patients with various pituitary tumors (23 prolactinomas, 49 nonfunctioning pituitary adenomas, 29 somatotroph adenomas, and five corticotroph tumors) and found that exosomal lncRNA H19 expression was significantly decreased in patients with pituitary tumors compared to healthy subjects. *In vitro*, exosomes facilitated the membrane transport of H19 which had an antiproliferative effect on GH3 cells. In a murine model, exosomal H19 inhibited the growth of distant GH3 tumors. Moreover, cabergoline increases H19 expression and acts synergistically with exosomal H19 to reduce GH3 cell proliferation. Finally, exosomal H19 inhibits 4E-Bp1 phosphorylation, thereby acting as a tumor suppressor. A higher exosomal H19 level has been linked to a better prognosis after dopamine agonist treatment. This study shows the potential of exosomally delivered lncRNA as a novel method for the treatment of dopamine-resistant prolactinomas and other pituitary tumors, particularly those resistant to available therapeutic options (86, 87).

## 7 Proteomic analysis of exosomes in pituitary adenomas

Epithelial markers, such as folate receptor 1 (FOLR1) and epithelial cell adhesion molecule (EPCAM), are overexpressed in pituitary adenoma tumor tissues and are generally known to be deeply connected with tumor malignancy and metastatic potential (88–91). Nevertheless, underexpression of these proteins and overexpression of mesenchymal markers such as vimentin or N-cadherin in pituitary adenoma tissue and exosomes may be related to epithelial-mesenchymal transition, which is positively correlated with pituitary tumor invasiveness.

Wang et al. compared the expression of FOLR1 and EPCAM in serum exosomes of invasive and non-invasive non-functional pituitary adenomas and found that they were significantly decreased in exosomes derived from the serum of invasive non-functional pituitary adenomas. This finding further suggests a role of epithelial-mesenchymal transition in the pathogenesis of invasive pituitary adenoma (92). These findings were further confirmed by Chen et al. in a study on epithelial-mesenchymal transition exosomal markers in 20 patients with pituitary (93).

In another study, Ren et al. found a positive correlation between the increased expression of matrix metalloproteinase-1 (MMP1) in exosomes derived from non-functioning pituitary adenomas and tumor invasiveness. MMP1 enrichment has been suggested as a promoter of tumor migration, growth, and angiogenesis through the protease-activated receptor-1 signaling pathway, causing disruption of the endothelial barrier (94).

Alterations in the expression of exosomal proteins indicate their potential as pioneering liquid biopsy markers of pituitary adenoma invasiveness.

## 8 Discussion

**Figure 2:**
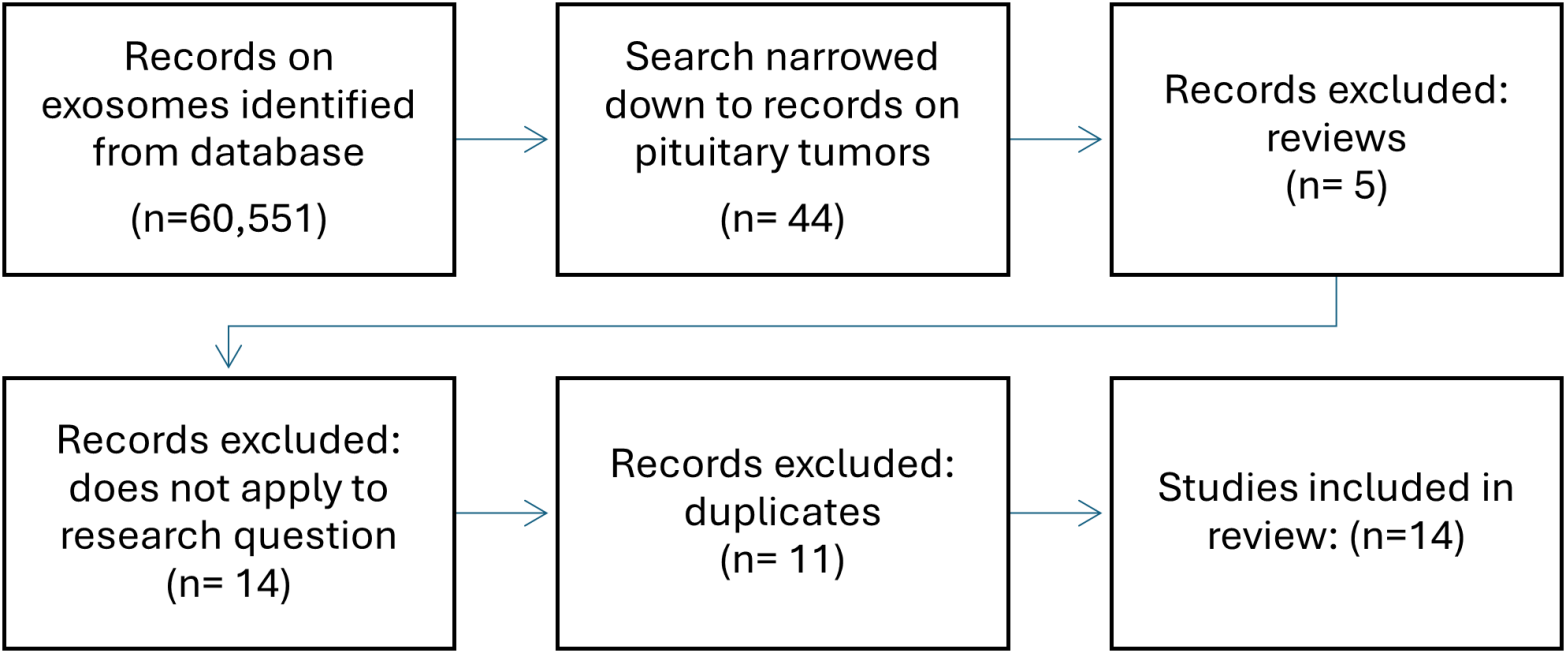
Flowchart of literature search.

Although study into the role of exosomes in pituitary tumors is still in its infancy, recently, their ability to reflect the functional state of the tumor, which has already been demonstrated in other neoplasms, has gained much attention. In a search in Pubmed using keywords, such as “pituitary adenoma, “pitnet”, “exosomes”, “exosomal” and “extracellular vesicles”, of the 60,551 search results on exosomes, 35 studies focused the pituitary gland and almost all were published in the previous five years (**Figure 2**). Due to their specific properties such as accessibility in serum and being able to carry cell-specific cargo including bioactive molecules exosomes have great potential in the search for biomarkers and new therapeutic methods. This review provides an overview of the current state of knowledge regarding the biology and function of exosomes in pituitary adenomas and their potential role in pituitary tumor diagnosis and treatment (**Table 2**). The results of the presented studies suggest that exosomal RNA is a possible biomarker for the diagnosis, progression, or recurrence of pituitary adenomas, laying the foundation for developing noninvasive techniques that are currently unavailable for patients with pituitary adenoma. This evidence also indicates the potential role of exosomes in epithelial mesenchymal transition related to pituitary adenoma invasiveness, which should be further explored. Functional studies have demonstrated the significant role of exosomal RNA in pituitary adenoma pathogenesis, tumorigenesis, and invasiveness as well as its potential application as a therapeutic agent. Despite the need to develop new diagnostic and therapeutic methods, there are weaknesses common to most of the presented studies: 1) regardless of advances in methods of isolation and characterization of exosomes, they remain difficult to evaluate mostly due to their low abundance in serum and possible cellular contamination, 2) most of the retrieved studies were based on relatively small groups of samples often with varying study designs and methods, 3) there is still little evidence on other factors potentially impacting exosomal release and composition such as a) ethnicity, b) sex, c) lifestyle, and d) co-morbidities, and last but not least, 4) generally studies focusing on pituitary adenomas are still scarce. No studies have been conducted on the role of epigenetic mechanisms or other molecules typically present in exosomes, and no studies concerning the role of exosomes in craniopharyngiomas are available.

**Table 2:** Functions of exosomal cargo in pituitary adenoma.

Pituitary adenoma (PA) type	Source material for exosomes	Molecule type	Main findings	References
Various	Plasma, tissue	miRNA	- Decreased miRNA expression in nonfunctioning PAs	Nemeth et al., 2019 (72)
Non-functioning	Plasma, tissue	miRNA	- Increased hsa-miR-486-5p expression (p=0.0006) - Candidate biomarker for progression or relapse of disease (AUC = 0.9432)	Lyu et al., 2022 (73)
Somatotroph adenoma	Serum	miRNA	-Decreased hsa-miR-320a, hsa-miR-423-5p expression (P < 0.001) -Antiproliferative effect of miR-423-5p on GH3 cells	Zhao et al., 2019 (74)
Various	Serum	circRNA	-circCCDC66 expression increased in PA (p < 0.01) -Candidate biomarker of PA with 80.00% sensitivity and 84.00% specificity	Yue et al., 2023 (76)
Non-functioning	Serum	mRNA	-*CDK6* and *RHOU* expression increased in invasive vs. non-invasive PA (p=0,0013, p=0,0052) -Markers of tumor invasiveness (AUC 0.772 and 0.757)	Yu et al.,2019 (77)
Non-functioning	Serum	mRNA	-*INSM1* mRNA expression increased in invasive PA (p < 0,05) -Marker of tumor invasiveness (AUC 0.719)	Bao et al., 2021 (78)
	GH3 and MMQ cell lines	miRNA	- miR-149-5p and miR-99a-3p inhibit tumor cell proliferation, migration and invasion	Zhao et al., 2021 (79)
Not specified	Tumor associated fibroblasts	circRNA	- circDennd1b expression increased in aggressive PA (p < 0,01)	Jiang et al., 2023 (80, 81)
	GH1 cell line	miRNA, mRNA	-Pituitary-derived exosomes attenuate malignant tumor cell migration, invasion and metastases	Zhou et al., 2022 (81)
	GH3 cell line	miRNA	-hsa-miR21-5p promotes abnormal bone formation in acromegaly	Xiong et al., 2020 (84)
Various	Whole blood	lncRNA	- lnc H19 expression decreased in pituitary tumor vs healthy subjects (p < 0,001) - anti-proliferative effect of H19 on GH3 cells	Zhang et al., 2019 (86)
Non-functioning	Serum	proteins	- FOLR1 and EPCAM expression decreased in invasive PA (p < 0,05) - Epithelial-mesenchymal transition markers indicate Pa invasiveness	Wang et al., 2019 (92)Chen et al., 2021 (93)
Non-functioning	Tissue	proteins	- MMP1 expression increased in invasive PA (p < 0,05) - MMP1 promotes tumor migration, growth and angiogenesis	Ren et al., 2022 (94)

Nevertheless, with technological progress, the role of exosomes in pituitary tumors is an area worthy of further exploration, which may bring about significant advances in their diagnosis and treatment.

## References

[1] Daly AF Beckers A. The epidemiology of pituitary adenomas. Endocrinol Metab Clin North Am. (2020) 49:347–55. doi: 10.1016/j.ecl.2020.04.002 32741475

[2] Tritos NA Miller KK. Diagnosis and management of pituitary adenomas: A review. JAMA. (2023) 329:1386–98. https://jamanetwork.com/journals/jama/fullarticle/2804060. 10.1001/jama.2023.5444 37097352

[3] Melmed S. Pituitary-tumor endocrinopathies. N Engl J Med. (2020) 382:937–50. https://pubmed.ncbi.nlm.nih.gov/32130815/. doi: 10.1056/NEJMra1810772 32130815

[4] Committee of Brain Tumor Registry of Japan Supported by the Japan Neurosurgical Society. Brain tumor registry of Japan (2005-2008). Neurol Med Chir (Tokyo). (2017) 57:9–102. doi: 10.2176/nmc.sup.2017-0001 28420810 PMC6760096

[5] Araujo-Castro M Berrocal VR Pascual-Corrales E. Pituitary tumors: epidemiology and clinical presentation spectrum. Hormones (Athens). (2020) 19:145–55. doi: 10.1007/s42000-019-00168-8 31933100

[6] Mete O Lopes MB. Overview of the 2017 WHO classification of pituitary tumors. Endocr Pathol. (2017) 28:228–43. doi: 10.1007/s12022-017-9498-z 28766057

[7] Asa SL Mete O Perry A Osamura RY. Overview of the 2022 WHO classification of pituitary tumors. Endocr Pathol. (2022) 33:6–26. doi: 10.1007/s12022-022-09703-7 35291028

[8] Casar-Borota O Burman P Lopes MB. The 2022 WHO classification of tumors of the pituitary gland: An update on aggressive and metastatic pituitary neuroendocrine tumors. Brain Pathol. (2024):e13302. doi: 10.1111/bpa.13302 39218431 PMC11669403

[9] Ho KKY Fleseriu M Wass J Katznelson L Raverot G Little AS. A proposed clinical classification for pituitary neoplasms to guide therapy and prognosis. Lancet Diabetes Endocrinol. (2024) 12:209–14. doi: 10.1016/S2213-8587(23)00382-0 PMC12051483 38301678

[10] Esposito D Olsson DS Ragnarsson O Buchfelder M Skoglund T Johannsson G. Non-functioning pituitary adenomas: indications for pituitary surgery and post-surgical management(2019) (Accessed 2024 Jul 30). 10.1007/s11102-019-00960-0 PMC6647426 31011999

[11] Zamanipoor Najafabadi AH van der Meulen M Zurita ALP Ahmed SF van Furth WR Charmandari E. Starting point for benchmarking outcomes and reporting of pituitary adenoma surgery within the European Reference Network on Rare Endocrine Conditions (Endo-ERN): results from a meta-analysis and survey study(2023) (Accessed 2024 Jul 30). 10.1530/EC-22-0349 PMC9782450 36327151

[12] Meij BP Lopes MBS Ellegala DB Alden TD Laws ER. The long-term significance of microscopic dural invasion in 354 patients with pituitary adenomas treated with transsphenoidal surgery. J Neurosurg [Internet]. (2002) 96:195–208. doi: 10.3171/jns.2002.96.2.0195 11838791

[13] Trouillas J Pascal R Sturm N Dantony E Cortet-Rudelli C Viennet G. A new prognostic clinicopathological classification of pituitary adenomas: a multicentric case-control study of 410 patients with 8 years post-operative follow-up(2013) (Accessed 2024 Oct 19). 10.1007/s00401-013-1084-y 23400299

[14] Dekkers OM Karavitaki N Pereira AM. The epidemiology of aggressive pituitary tumors (and its challenges). Rev Endocr Metab Disord [Internet]. (2020) 21:209–12. doi: 10.1007/s11154-020-09556-7 PMC7303064 32361816

[15] Ho KKY Gadelha M Kaiser UB Reincke M Melmed S. The NETting of pituitary adenoma: a gland illusion. Pituitary [Internet]. (2022) 25:349–51. doi: 10.1007/s11102-022-01235-x PMC9170656 35616761

[16] McCormack A Dekkers OM Petersenn S Popovic V Trouillas J Raverot G. Treatment of aggressive pituitary tumors and carcinomas: results of a European Society of Endocrinology (ESE) survey 2016. Eur J Endocrinol. (2018) 178:265–76. doi: 10.1530/EJE-17-0933 29330228

[17] Rezaie J Feghhi M Etemadi T. A review on exosomes application in clinical trials: perspective, questions, and challenges. Cell Communication Signaling. (2022) 20:1–13. doi: 10.1186/s12964-022-00959-4 36123730 PMC9483361

[18] Perocheau D Touramanidou L Gurung S Gissen P Baruteau J. Clinical applications for exosomes: Are we there yet. Br J Pharmacol. (2021) 178:2375. https://pmc.ncbi.nlm.nih.gov/articles/PMC8432553/. 33751579 10.1111/bph.15432 PMC8432553

[19] Batista IA MaChado JC Melo SA. Advances in exosomes utilization for clinical applications in cancer. Trends Cancer. (2024) 10:947–68. doi: 10.1016/j.trecan.2024.07.010 39168775

[20] Wolf P. The nature and significance of platelet products in human plasma. Br J Hematol. (1967) 13:269–88. doi: 10.1111/j.1365-2141.1967.tb08741.x 6025241

[21] Yáñez-Mó M Siljander PRM Andreu Z Zavec AB Borràs FE Buzas EI. Biological properties of extracellular vesicles and their physiological functions. J Extracell Vesicles. (2015) 4:27066. doi: 10.3402/jev.v4.27066 25979354 PMC4433489

[22] He M Zeng Y. Microfluidic exosome analysis toward liquid biopsy for cancer. J Lab Autom. (2016) 21:599–608. doi: 10.1177/2211068216651035 27215792 PMC5556932

[23] Ratajczak J Miekus K Kucia M Zhang J Reca R Dvorak P. Embryonic stem cell-derived microvesicles reprogram hematopoietic progenitors: evidence for horizontal transfer of mRNA and protein delivery. Leukemia. (2006) 20:847–56. https://www.nature.com/articles/2404132. 10.1038/sj.leu.2404132 16453000

[24] Valadi H Ekström K Bossios A Sjöstrand M Lee JJ Lötvall JO. Exosome-mediated transfer of mRNAs and microRNAs is a novel mechanism of genetic exchange between cells. Nat Cell Biol. (2007) 9:654–9. doi: 10.1038/ncb1596 17486113

[25] Hefley BS Deighan C Vasini B Khan A Hjortdal J Riaz KM. Revealing the presence of tear extracellular vesicles in Keratoconus. Exp Eye Res. (2022), 224:109242. doi: 10.1016/j.exer.2022.109242 36084727 PMC10159047

[26] Hauser P Wang S Didenko VV. Apoptotic bodies: selective detection in extracellular vesicles. Methods Mol Biol. (2017) 1554:193–200. doi: 10.1007/978-1-4939-6759-9_12 28185192

[27] Cloos AS Ghodsi M Stommen A Vanderroost J Dauguet N Pollet H. Interplay between plasma membrane lipid alteration, oxidative stress and calcium-based mechanism for extracellular vesicle biogenesis from erythrocytes during blood storage. Front Physiol. (2020) 11:712. doi: 10.3389/fphys.2020.00712 32719614 PMC7350142

[28] D’Angelo G Raposo G Nishimura T Suetsugu S. Protrusion-derived vesicles: new subtype of EVs? Nat Rev Mol Cell Biol. (2022) 24:2. https://www.nature.com/articles/s41580-022-00555-x. 10.1038/s41580-022-00555-x 36280788

[29] Hu HT Nishimura T Kawana H Dante RAS D’Angelo G Suetsugu S. The cellular protrusions for inter-cellular material transfer: similarities between filopodia, cytonemes, tunneling nanotubes, viruses, and extracellular vesicles(2024) (Accessed 2024 Oct 18). 10.3389/fcell.2024.1422227 PMC11257967 39035026

[30] Nishimura T Oyama T Hu HT Fujioka T Hanawa-Suetsugu K Ikeda K. Filopodium-derived vesicles produced by MIM enhance the migration of recipient cells. Dev Cell. (2021) 56:842–859.e8. http://www.cell.com/article/S1534580721001696/fulltext. 33756122 10.1016/j.devcel.2021.02.029

[31] Zhang Y Liu Y Liu H Tang WH. Exosomes: biogenesis, biologic function and clinical potential. Cell Biosci. (2019) 9:19. doi: 10.1186/s13578-019-0282-2 30815248 PMC6377728

[32] Han QF Li WJ Hu KS Gao J Zhai WL Yang JH. Exosome biogenesis: machinery, regulation, and therapeutic implications in cancer. Mol Cancer. (2022) 21:1. doi: 10.1186/s12943-022-01671-0 36320056 PMC9623991

[33] Kalluri R LeBleu VS. The biology, function, and biomedical applications of exosomes(2020) (Accessed 2024 Jul 30). 10.1126/science.aau6977 PMC7717626 32029601

[34] Gurung S Perocheau D Touramanidou L Baruteau J. The exosome journey: from biogenesis to uptake and intracellular signaling(2021) (Accessed 2024 Oct 15). 10.1186/s12964-021-00730-1 PMC8063428 33892745

[35] Guescini M Genedani S Stocchi V Agnati LF. Astrocytes and Glioblastoma cells release exosomes carrying mtDNA(2010) (Accessed 2024 Oct 16). 10.1007/s00702-009-0288-8 19680595

[36] Thakur BK Zhang H Becker A Matei I Huang Y Costa-Silva B. Double-stranded DNA in exosomes: a novel biomarker in cancer detection. Cell Res. (2014) 24:6. https://www.nature.com/articles/cr201444. 10.1038/cr.2014.44 PMC4042169 24710597

[37] Balaj L Lessard R Dai L Cho YJ Pomeroy SL Breakefield XO. Tumor microvesicles contain retrotransposon elements and amplified oncogene sequences(2011) (Accessed 2024 Oct 16). 10.1038/ncomms1180 PMC3040683 21285958

[38] Yoshioka Y Konishi Y Kosaka N Katsuda T Kato T Ochiya T. Comparative marker analysis of extracellular vesicles in different human cancer types. J Extracell Vesicles. (2013) 2. doi: 10.3402/jev.v2i0.20424 PMC3760642 24009892

[39] Feng D Zhao WL Ye YY Bai XC Liu RQ Chang LF. Cellular internalization of exosomes occurs through phagocytosis. Traffic. (2010) 11:675–87. doi: 10.1111/j.1600-0854.2010.01041.x 20136776

[40] Salomon C Das S Erdbrügger U Kalluri R Kiang Lim S Olefsky JM. Extracellular vesicles and their emerging roles as cellular messengers in endocrinology: an endocrine society scientific statement. Endocr Rev. (2022) 43:441–68. doi: 10.1210/endrev/bnac009 PMC10686249 35552682

[41] Welsh JA Goberdhan DCI O’Driscoll L Buzas EI Blenkiron C Bussolati B. Minimal information for studies of extracellular vesicles (MISEV2023): From basic to advanced approaches. J Extracell Vesicles. (2024) 13. doi: 10.1002/jev2.12404 PMC10850029 38326288

[42] Kim J Shin H Kim J Kim J Park J. Isolation of high-purity extracellular vesicles by extracting proteins using aqueous two-phase system. PloS One. (2015) 10:e0129760. doi: 10.1371/journal.pone.0129760 26090684 PMC4475045

[43] George JN Thoi LL McManus LM Reimann TA. Isolation of human platelet membrane microparticles from plasma and serum. Blood. (1982) 60:834–40. doi: 10.1182/blood.V60.4.834.834 7115953

[44] Witwer KW Buzás EI Bemis LT Bora A Lässer C Lötvall J. Standardization of sample collection, isolation and analysis methods in extracellular vesicle research. J Extracell Vesicles. (2013) 2. doi: 10.3402/jev.v2i0.20360 PMC3760646 24009894

[45] Zhang Y Bi J Huang J Tang Y Du S Li P. Exosome: A review of its classification, isolation techniques, storage, diagnostic and targeted therapy applications. Int J Nanomedicine. (2020) 15:6917–34. doi: 10.2147/IJN.S264498 PMC7519827 33061359

[46] Gardiner C Di VD Sahoo S Théry C Witwer KW Wauben M. Techniques used for the isolation and characterization of extracellular vesicles: results of a worldwide survey(2016) (Accessed 2024 Jul 30). 10.3402/jev.v5.32945 PMC5090131 27802845

[47] Théry C Witwer KW Aikawa E Alcaraz MJ Anderson JD Andriantsitohaina R. Minimal information for studies of extracellular vesicles 2018 (MISEV2018): a position statement of the International Society for Extracellular Vesicles and update of the MISEV2014 guidelines(2018) (Accessed 2024 Jul 30). 10.1080/20013078.2018.1535750 PMC6322352 30637094

[48] Hoshino A Kim HS Bojmar L Gyan KE Cioffi M Hernandez J. Extracellular vesicle and particle biomarkers define multiple human cancers. Cell. (2020) 182:1044–61. doi: 10.1016/j.cell.2020.07.009 PMC7522766 32795414

[49] Kim CY Baek S Cha J Yang S Kim E Marcotte EM. HumanNet v3: an improved database of human gene networks for disease research(2022) (Accessed 2024 Jul 30). 10.1093/nar/gkab1048 PMC8728227 34747468

[50] Kalra H Simpson RJ Ji H Aikawa E Altevogt P Askenase P. Vesiclepedia: a compendium for extracellular vesicles with continuous community annotation. PloS Biol. (2012) 10:e1001450. doi: 10.1371/journal.pbio.1001450 23271954 PMC3525526

[51] Keerthikumar S Chisanga D Ariyaratne D Al Saffar H Anand S Zhao K. ExoCarta: A web-based compendium of exosomal cargo. J Mol Biol. (2016) 428:688–92. doi: 10.1016/j.jmb.2015.09.019 PMC4783248 26434508

[52] EVpedia - Extracellular vesicle database for high-throughput data and publications. Available online at: https://evpedia.info/evpedia2_xe/ (Accessed October 15, 2024).

[53] Vesiclepedia: Home - Extracellular vesicles database. Available online at: http://www.microvesicles.org/ (Accessed 2024 Oct 15).

[54] ExoCarta: Home - Exosome database. Available online at: http://www.exocarta.org/ (Accessed 2024 Oct 15).

[55] Li W Li C Zhou T Liu X Liu X Li X. Role of exosomal proteins in cancer diagnosis. Mol Cancer [Internet]. (2017) 16:145. doi: 10.1186/s12943-017-0706-8 28851367 PMC5576100

[56] Kok VC Yu CC. Cancer-derived exosomes: their role in cancer biology and biomarker development. Int J Nanomedicine. (2020) 15:8019–36. doi: 10.2147/IJN.S272378 PMC7585279 33116515

[57] Lea J Sharma R Yang F Zhu H Sally Ward E Schroit AJ. Detection of phosphatidylserine-positive exosomes as a diagnostic marker for ovarian Malignancies: a proof of concept study. Oncotarget. (2017) 8:14395–407. doi: 10.18632/oncotarget.14795 PMC5362413 28122335

[58] Stevic I Müller V Weber K Fasching PA Karn T Marmé F. Specific microRNA signatures in exosomes of triple-negative and HER2-positive breast cancer patients undergoing neoadjuvant therapy within the GeparSixto trial. BMC Med. (2018) 16:179. doi: 10.1186/s12916-018-1163-y 30301470 PMC6178264

[59] Skotland T Ekroos K Kauhanen D Simolin H Seierstad T Berge V. Molecular lipid species in urinary exosomes as potential prostate cancer biomarkers. Eur J Cancer. (2017) 70:122–32. doi: 10.1016/j.ejca.2016.10.011 27914242

[60] Thakur BK Zhang H Becker A Matei I Huang Y Costa-Silva B. Double-stranded DNA in exosomes: a novel biomarker in cancer detection. Cell Res. (2014) 24:766–9. doi: 10.1038/cr.2014.44 PMC4042169 24710597

[61] Fuhrmann G Serio A Mazo M Nair R Stevens MM. Active loading into extracellular vesicles significantly improves the cellular uptake and photodynamic effect of porphyrins. J Control Release. (2015) 205:35–44. doi: 10.1016/j.jconrel.2014.11.029 25483424

[62] Haney MJ Klyachko NL Zhao Y Gupta R Plotnikova EG He Z. Exosomes as drug delivery vehicles for Parkinson’s disease therapy. J Control Release. (2015) 207:18–30. doi: 10.1016/j.jconrel.2015.03.033 25836593 PMC4430381

[63] Wang P Wang H Huang Q Peng C Yao L Chen H. Exosomes from M1-polarized macrophages enhance paclitaxel antitumor activity by activating macrophages-mediated inflammation. Theranostics [Internet]. (2019) 9:1714–27. doi: 10.7150/thno.30716 PMC6485189 31037133

[64] Home | ClinicalTrials.gov . Available online at: https://clinicaltrials.gov/ (Accessed 2024 Oct 15).

[65] Lu TX Rothenberg ME. MicroRNA. J Allergy Clin Immunol. (2018) 141:1202–7. doi: 10.1016/j.jaci.2017.08.034 PMC5889965 29074454

[66] Cui M Wang H Yao X Zhang D Xie Y Cui R. Circulating microRNAs in cancer: potential and challenge. Front Genet. (2019) 10:626. doi: 10.3389/fgene.2019.00626 31379918 PMC6656856

[67] Arroyo JD Chevillet JR Kroh EM Ruf IK Pritchard CC Gibson DF. Argonaute2 complexes carry a population of circulating microRNAs independent of vesicles in human plasma. Proc Natl Acad Sci U S A. (2011) 108:5003–8. doi: 10.1073/pnas.1019055108 PMC3064324 21383194

[68] Tian F Shen Y Chen Z Li R Ge Q. No Significant Difference between Plasma miRNAs and Plasma-Derived Exosomal miRNAs from Healthy People. BioMed Res Int. (2017) 2017:1304816. https://www.ncbi.nlm.nih.gov/pmc/articles/PMC5471588/. 28656135 10.1155/2017/1304816 PMC5471588

[69] Zhou X Wen W Shan X Zhu W Xu J Guo R. A six-microRNA panel in plasma was identified as a potential biomarker for lung adenocarcinoma diagnosis. Oncotarget. (2016) 8:6513–25. doi: 10.18632/oncotarget.14311 PMC5351649 28036284

[70] Huang Z Zhu D Wu L He M Zhou X Zhang L. Six serum-based miRNAs as potential diagnostic biomarkers for gastric cancer. Cancer Epidemiol Biomarkers Prev. (2017) 26:188–96. doi: 10.1158/1055-9965.EPI-16-0607 27756776

[71] Zhang B Pan X Cobb GP Anderson TA. microRNAs as oncogenes and tumor suppressors. Dev Biol. (2007) 302:1–12. doi: 10.1016/j.ydbio.2006.08.028 16989803

[72] Németh K Darvasi O Likó I Szücs N Czirják S Reiniger L. Comprehensive Analysis of Circulating miRNAs in the Plasma of Patients With Pituitary Adenomas. J Clin Endocrinol Metab. (2019) 104:4151–68. https://pubmed.ncbi.nlm.nih.gov/31112271/. doi: 10.1210/jc.2018-02479 31112271

[73] Lyu L Li H Chen C Yu Y Wang L Yin S. Exosomal miRNA profiling is a potential screening route for non-functional pituitary adenoma(2022) (Accessed 2024 Jul 30). 10.3389/fcell.2021.771354 PMC8804500 35118066

[74] Zhao S Li J Feng J Li Z Liu Q Lv P. Identification of Serum miRNA-423-5p Expression Signature in Somatotroph Adenomas. Int J Endocrinol [Internet]. (2019) 2019:8516858. https://www.ncbi.nlm.nih.gov/pmc/articles/PMC6662485/. 31391849 10.1155/2019/8516858 PMC6662485

[75] Zhao X Zhong Y Wang X Shen J An W. Advances in circular RNA and its applications. Int J Med Sci. (2022) 19:975–85. https://www.ncbi.nlm.nih.gov/pmc/articles/PMC9254372/. 10.7150/ijms.71840 PMC9254372 35813288

[76] Yue X Lan F Liu W. Serum exosomal circCCDC66 as a potential diagnostic and prognostic biomarker for pituitary adenomas. Front Oncol [Internet]. (2023) 13:1268778. https://www.ncbi.nlm.nih.gov/pmc/articles/PMC10720038/. 38098508 10.3389/fonc.2023.1268778 PMC10720038

[77] Yu S Wang X Cao K Bao X Yu J. Identification of CDK6 and RHOU in serum exosome as biomarkers for the invasiveness of non-functioning pituitary adenoma. Chin Med Sci J. (2019) 34:168–76. doi: 10.24920/003585 31601299

[78] Bao X Wang G Yu S Sun J He L Zhao H. Transcriptomic analysis identifies a tumor subtype mRNA classifier for invasive non-functioning pituitary neuroendocrine tumor diagnostics(2021) (Accessed 2024 Jul 30). 10.7150/thno.47525 PMC7681103 33391466

[79] Zhao P Cheng J Li B Nie D Li C Gui S. Up-regulation of the expressions of MiR-149-5p and MiR-99a-3p in exosome inhibits the progress of pituitary adenomas(2021) (Accessed 2024 Jul 30). 10.1007/s10565-020-09570-0 33400021

[80] Jiang Q Lei Z Wang Z Wang Q Zhang Z Liu X. Tumor-Associated Fibroblast-Derived Exosomal circDennd1b Promotes Pituitary Adenoma Progression by Modulating the miR-145-5p/ONECUT2 Axis and Activating the MAPK Pathway(2023) (Accessed 2024 Jul 30). 10.3390/cancers15133375 PMC10340501 37444485

[81] Zhou C Shen S Moran R Deng N Marbán E Melmed S. Pituitary somatotroph adenoma-derived exosomes: characterization of nonhormonal actions. J Clin Endocrinol Metab. (2022) 107:379–97. doi: 10.1210/clinem/dgab651 PMC8764361 34467411

[82] Vulpis E Cecere F Molfetta R Soriani A Fionda C Peruzzi G. Genotoxic stress modulates the release of exosomes from multiple myeloma cells capable of activating NK cell cytokine production: Role of HSP70/TLR2/NF-kB axis(2017) (Accessed 2024 Jul 30). 10.1080/2162402X.2017.1279372 PMC5384384 28405503

[83] Hedlund M Nagaeva O Kargl D Baranov V Mincheva-Nilsson L. Thermal- and oxidative stress causes enhanced release of NKG2D ligand-bearing immunosuppressive exosomes in leukemia/lymphoma T and B cells. PloS One. (2011) 6:e16899. doi: 10.1371/journal.pone.0016899 21364924 PMC3045385

[84] Xiong Y Tang Y Fan F Zeng Y Li C Zhou G. Exosomal hsa-miR-21-5p derived from growth hormone-secreting pituitary adenoma promotes abnormal bone formation in acromegaly. Transl Res. (2020) 215:1–16. doi: 10.1016/j.trsl.2019.07.013 31469974

[85] Xia Y Pei T Zhao J Wang Z Shen Y Yang Y. Long noncoding RNA H19: functions and mechanisms in regulating programmed cell death in cancer. Cell Death Discovery [Internet]. (2024) 10:76. doi: 10.1038/s41420-024-01832-8 38355574 PMC10866971

[86] Zhang Y Liu YT Tang H Xie WQ Yao H Gu WT. Exosome-transmitted lncRNA H19 inhibits the growth of pituitary adenoma. J Clin Endocrinol Metab. (2019) 104:6345–56. doi: 10.1210/jc.2019-00536 31369093

[87] Wu ZR Yan L Liu YT Cao L Guo YH Zhang Y. Inhibition of mTORC1 by lncRNA H19 via disrupting 4E-BP1/Raptor interaction in pituitary tumors. Nat Commun. (2018) 9:4624. doi: 10.1038/s41467-018-06853-3 30397197 PMC6218470

[88] Fagotto F. EpCAM as modulator of tissue plasticity(2020) (Accessed 2024 Oct 15). 10.3390/cells9092128 PMC7563481 32961790

[89] Nawaz FZ Kipreos ET. Emerging roles for folate receptor FOLR1 in signaling and cancer(2022) (Accessed 2024 Oct 15). 10.1016/j.tem.2021.12.003 PMC8923831 35094917

[90] Chen X Pang B Liang Y Xu SC Xin T Fan HT. Overexpression of EpCAM and Trop2 in pituitary adenomas(2014) (Accessed 2024 Oct 15). PMC4270592 25550831

[91] Liu X Ma S Yao Y Li G Feng M Deng K. Differential expression of folate receptor alpha in pituitary adenomas and its relationship to tumor behavior(2012) (Accessed 2024 Oct 15). 10.1227/NEU.0b013e3182417e76 22089756

[92] Wang H Chen K Yang Z Li W Wang C Zhang G. Diagnosis of invasive nonfunctional pituitary adenomas by serum extracellular vesicles. Anal Chem. (2019) 91:9580–9. doi: 10.1021/acs.analchem.9b00914 31264409

[93] Chen K Li G Kang X Liu P Qian L Shi Y. EMT-related markers in serum exosomes are potential diagnostic biomarkers for invasive pituitary adenomas. Neuropsychiatr Dis Treat. (2021) 17:3769–80. doi: 10.2147/NDT.S339067 PMC8711285 34992371

[94] Ren Y Wang Y Bao X Feng M Xing B Lian W. Diagnosis of invasive non-functional pituitary adenomas using exosomal biomarkers(2022) (Accessed 2024 Jul 30). 10.1016/j.cca.2022.01.014 35085587

